# Association of Adverse Childhood Experiences with Fear of Childbirth and Prenatal Attachment Among Pregnant Women: A Cross-Sectional Study

**DOI:** 10.3390/healthcare14213662

**Published:** 2026-10-28

**Authors:** Arzu Aydoğan, Meserret Aslan, Fadime Bayrı Bingöl, Ayfer Öztünç İsi, Tuğba Topcu, Nuran Temiz

**Affiliations:** 1 Department of Psychiatric Nursing, Faculty of Health Sciences, Yuksek Ihtisas University, 06790 Ankara, Türkiye; arzu.aydogan@yuksekihtisas.edu.tr; 2 Department of Midwifery, Faculty of Health Sciences, Istanbul Atlas University, 34408 Istanbul, Türkiye; 3 Department of Midwifery, Faculty of Health Sciences, Marmara University, 34854 Istanbul, Türkiye; fadime.bingol@marmara.edu.tr; 4 Department of Midwifery, Faculty of Health Sciences, Istanbul Medipol University, 34815 Istanbul, Türkiye; ayfer.oztunc@medipol.edu.tr; 5 Marmara University Hospital, 34899 Istanbul, Türkiye; topcutugba65@gmail.com; 6 Tuzla State Hospital, Ministry of Health, 34947 Istanbul, Türkiye; nrantemiz@gmail.com

**Keywords:** adverse childhood experiences, fear of childbirth, prenatal attachment, pregnancy, trauma-informed care

## Abstract

**Highlights:**

**What are the main findings?**

• Higher adverse childhood experience scores were associated with greater fear of childbirth after adjustment for measured sociodemographic and obstetric characteristics.
• Adverse childhood experiences were not significantly associated with prenatal attachment after adjustment; associations involving educational level and pregnancy planning status were identified in secondary exploratory analyses.

**What are the implications of the main findings?**

• The findings support consideration of trauma-informed, privacy-sensitive approaches to antenatal care but do not establish the clinical benefits of routine adverse childhood experience screening.
• Prenatal attachment should be considered within a broader psychosocial context, including educational background and pregnancy planning status.

**Abstract:**

**Background/Objectives:** Adverse childhood experiences (ACEs) may be associated with psychological responses during pregnancy. This study examined the associations of ACEs with fear of childbirth and prenatal attachment, accounting for measured sociodemographic and obstetric characteristics. **Methods:** This cross-sectional study included 537 pregnant women attending an obstetrics and gynecology outpatient clinic in Istanbul, Türkiye, between June and December 2022. Data were collected using a Personal Information Form, the Adverse Childhood Experiences Scale, the Wijma Delivery Expectancy/Experience Questionnaire-A (W-DEQ-A), and the Prenatal Attachment Inventory (PAI). Analyses included group comparisons, Spearman correlations, and multivariable regression with sensitivity analyses. **Results**: The mean maternal age was 28.1 ± 4.47 years; 43.8% had a university education, 36.9% were employed, and 74.3% reported a planned pregnancy. Higher ACE scores were associated with greater fear of childbirth in the fully adjusted model (B = 2.430, β = 0.154, 95% CI: 1.102–3.758, *p* < 0.001), with a consistent association in the minimally adjusted model. ACE scores were not significantly associated with prenatal attachment in the fully adjusted model (B = 0.375, β = 0.054, *p* = 0.206). In secondary exploratory analyses, unplanned pregnancy was associated with lower PAI scores compared with planned pregnancy (B = −3.905, 95% CI: −5.855–−1.955, *p* < 0.001). **Conclusions**: Higher ACE scores were associated with greater fear of childbirth, whereas no statistically significant association with prenatal attachment was observed after adjustment. These findings support consideration of trauma-informed antenatal care but do not establish causality or demonstrate the benefit of routine ACE screening.

## 1. Introduction

Pregnancy involves substantial biological and psychological adaptation. Experiences from earlier life may acquire renewed relevance during pregnancy and may shape how women perceive uncertainty, bodily change, and the anticipated birth process. Adverse childhood experiences (ACEs) comprise cumulative exposures to abuse, neglect, and household dysfunction and have been associated with a broad range of adult physical and mental health outcomes [1,2]. Beyond their individual consequences, ACEs remain a significant public health and social concern, as experiences of childhood adversity and victimization may show continuity across generations. Previous research examining three generations has highlighted the potential intergenerational transmission of physical, emotional, and sexual victimization [3]. During pregnancy, ACEs may therefore represent an important psychosocial context for understanding emotional responses to childbirth and the developing relationship with the fetus [4].

Fear of childbirth is a multidimensional experience that may involve concerns about pain, loss of control, medical interventions, injury, and harm to the mother or infant. Previous studies have reported positive associations between childhood trauma and fear of childbirth [5,6,7,8]. Trauma-informed maternity care frameworks further suggest that birth-related procedures, exposure, and perceived loss of control may be particularly salient for women with earlier traumatic experiences [6]. However, fear of childbirth is also associated with obstetric and sociodemographic characteristics, and the independent contribution of ACEs should therefore be evaluated in multivariable models.

Prenatal attachment describes the emotions, thoughts, and behaviors through which a pregnant woman relates to her fetus. Childhood adversity may plausibly be associated with prenatal attachment through attachment patterns, maternal mental health, perceived safety, or other psychosocial pathways. Nevertheless, findings are inconsistent. Some studies have reported associations between childhood trauma and maternal-fetal attachment, whereas others suggest that the relationship may be weak or dependent on intervening psychological factors [4,9,10,11]. Maternal education, social resources, and whether the pregnancy was planned may also be relevant to prenatal attachment [12,13,14].

Existing studies have more often examined ACEs in relation to fear of childbirth or prenatal attachment separately. Evidence evaluating both outcomes within the same sample and simultaneously accounting for relevant sociodemographic and obstetric characteristics remains limited. Examining both outcomes within a common analytical framework may contribute to a more integrated understanding of psychosocial vulnerability during pregnancy.

Accordingly, this study aimed to examine the associations of ACEs with fear of childbirth and prenatal attachment among pregnant women. We hypothesized that higher ACE scores would be associated with higher fear-of-childbirth scores and, secondarily, with lower prenatal attachment scores.

## 2. Materials and Methods

### 2.1. Study Design and Setting

This cross-sectional study was conducted at the Obstetrics and Gynecology Outpatient Clinic of Marmara University Pendik Training and Research Hospital, Istanbul, Türkiye. Data were collected between 10 June and 31 December 2022 from pregnant women attending routine antenatal follow-up.

### 2.2. Participants and Sampling

The study population comprised pregnant women attending the outpatient clinic during the prespecified data-collection period. Participants were recruited using a nonprobability convenience sampling approach. Eligibility was assessed before recruitment, and only women who met the predefined eligibility criteria were invited to participate. The original study protocol specified a minimum sample size of 376 participants. A total of 537 eligible pregnant women voluntarily agreed to participate and were included in the study, exceeding the protocol-defined minimum sample size. No post hoc power calculation was performed to justify the achieved sample size.

The inclusion criteria were being aged 18–45 years, being able to read and understand Turkish, voluntarily agreeing to participate, living with a spouse, and having no self-reported history of clinically significant pregnancy, childbirth, or postpartum complications in previous pregnancies. Women reporting clinically significant maternal or fetal complications in the current pregnancy were also considered ineligible. These criteria were assessed before study invitation; therefore, women who did not meet the eligibility requirements were not recruited rather than being excluded after enrollment. The number of women who did not meet the eligibility criteria was not systematically recorded.

Information regarding previous and current obstetric complications was obtained through participant self-report and was not independently verified against medical records. The restriction concerning previous obstetric complications was established to reduce the potential influence of prior difficult or traumatic obstetric experiences on current fear-of-childbirth scores. The requirement to live with a spouse was part of the original study protocol. The potential implications of these eligibility restrictions for selection bias and generalizability are addressed in the Strengths and Limitations section.

### 2.3. Measures

#### 2.3.1. Personal Information Form

A researcher-developed form assessed maternal age, educational level, employment status, economic status, family type, gestational age, pregnancy and birth history, and pregnancy planning status.

#### 2.3.2. Adverse Childhood Experiences Scale

The 10-item Adverse Childhood Experiences Scale assesses cumulative exposure to childhood adversity, including abuse, neglect, and household dysfunction. Each item is scored dichotomously as 0 (absence of the experience) or 1 (presence of the experience), yielding a cumulative total score ranging from 0 to 10, with higher scores indicating exposure to a greater number of adverse childhood experiences. The Turkish validity and reliability study was conducted by Gündüz et al. [15]. In the present sample, Cronbach’s alpha was 0.641. This finding was interpreted cautiously because the ACE scale represents a cumulative index of conceptually heterogeneous experiences rather than a unidimensional measure of a single psychological construct. Consequently, internal consistency alone may not fully reflect the measurement properties of the ACE index. The continuous ACE total score was used in correlation and regression analyses, while participants were categorized into three groups (0, 1–3, and ≥4 ACEs) for group comparisons. The ≥4 threshold was selected as an indicator of high cumulative adversity exposure, consistent with previous ACE research [1].

#### 2.3.3. Wijma Delivery Expectancy/Experience Questionnaire-A

The W-DEQ-A assesses fear and expectations related to childbirth and consists of 33 items scored from 0 to 5. Positively formulated items 2, 3, 6, 7, 8, 11, 12, 15, 19, 20, 24, 25, 27, and 31 were reverse-scored before summation. Total scores range from 0 to 165, with higher scores indicating greater fear of childbirth. The Turkish validation study reported a Cronbach’s alpha of 0.89 [16]. In the present study, Cronbach’s alpha was 0.874.

#### 2.3.4. Prenatal Attachment Inventory

The Prenatal Attachment Inventory (PAI) contains 21 items assessing maternal feelings, thoughts, and behaviors toward the fetus. Items are scored from 1 to 4 and summed without reverse scoring, producing a total score of 21–84; higher scores indicate greater prenatal attachment. The Turkish adaptation reported a Cronbach’s alpha of 0.84 [17]. Cronbach’s alpha was 0.891 in the present sample.

### 2.4. Data Collection

Eligible pregnant women were informed about the purpose and scope of the study before providing voluntary informed consent. Data were collected through face-to-face interviews in a private setting before routine clinical examinations. The researchers verbally administered the questionnaires and recorded participants’ responses on the study forms. Particular attention was given to maintaining privacy and confidentiality during the administration of sensitive ACE items. Participants were explicitly informed that they could decline to answer any question they felt uncomfortable with, without any consequences for their participation or clinical care. Nevertheless, all 537 participants provided complete responses to all 10 ACE items, and no item-level missing data were observed.

### 2.5. Ethical Considerations

The study was conducted in accordance with the Declaration of Helsinki and was approved by the Marmara University Faculty of Health Sciences Non-Interventional Clinical Research Ethics Committee (protocol code 58; 28 April 2022). Administrative permission to conduct the study in the relevant health institution was granted by the Istanbul Provincial Health Directorate, Research, Printed Publication and Announcement Content Evaluation Committee (decision no. 2022/12; 9 June 2022).

### 2.6. Statistical Analysis

All statistical analyses were performed using IBM SPSS Statistics version 32.0 (IBM Corp., Armonk, NY, USA). Before analysis, the dataset was screened for missing values, coding inconsistencies, and observations outside the theoretical ranges of the study measures. Potentially erroneous or clinically implausible values were checked against the original data collection forms and corrected only when the source information could be verified. No values were inferred or replaced without source verification. Following data cleaning, all 537 participants had complete data for the variables included in the primary analyses, and no imputation was performed. Adverse childhood experience (ACE) total scores were calculated by summing the 10 dichotomously scored items. All participants completed every ACE item; therefore, no missing-item imputation or prorating was necessary. Gestational age was recorded in completed weeks.

Continuous variables were summarized using means and standard deviations or medians and interquartile ranges, as appropriate, whereas categorical variables were presented as frequencies and percentages. Between-group comparisons were performed using independent-samples *t*-tests or Mann–Whitney U tests for two groups, and one-way analysis of variance (ANOVA), Welch ANOVA, or Kruskal–Wallis tests for three or more groups, depending on distributional characteristics and homogeneity of variance. Games–Howell post hoc tests were applied following significant Welch ANOVA results. Effect sizes were reported as rank-biserial correlations for Mann–Whitney U tests, omega squared (ω^2^) for parametric comparisons involving three or more groups, epsilon squared (ε^2^) for Kruskal–Wallis tests, and Hedges’ g for pairwise post hoc comparisons. Associations among gestational age, ACE total scores, fear-of-childbirth scores (W-DEQ-A), and prenatal attachment scores (PAI) were examined using Spearman rank correlations. Group comparisons and correlation analyses were considered exploratory and were not used to determine covariate inclusion in the multivariable models. No formal adjustment for multiple testing was applied to these exploratory analyses; therefore, findings were interpreted cautiously, with emphasis on effect sizes and the overall consistency of associations rather than isolated *p*-values.

The primary analyses consisted of two multivariable linear regression models, with W-DEQ-A and PAI total scores as the respective dependent variables. Both models included ACE total score, maternal age, gestational age, parity, educational level, employment status, economic status, and pregnancy planning status. Parity was dichotomized as nulliparity or multiparity based on reported previous birth history. Covariates were selected a priori according to their conceptual and literature-based relevance rather than univariable statistical significance. Maternal age and parity were included to account for reproductive characteristics and childbirth-related expectations, while gestational age was included to account for differences across pregnancy. Educational level, employment status, and economic status were considered indicators of socioeconomic circumstances potentially related to maternal psychological experiences. Pregnancy planning status was included because pregnancy intendedness may be associated with maternal emotional responses and prenatal attachment. The same covariates were retained in both models to ensure analytical consistency. The regression analyses were intended to estimate associations conditional on measured covariates rather than establish causal effects.

To examine the potential influence of adjustment for variables that may occur downstream of childhood adversity, minimally adjusted models including ACE total score, maternal age, and gestational age were fitted for both W-DEQ-A and PAI outcomes. These models were compared with the fully adjusted models, which additionally included parity, educational level, employment status, economic status, and pregnancy planning status. The comparison was intended to evaluate the sensitivity of the ACE coefficient to covariate adjustment, rather than to identify a definitive causal effect. HC3 heteroskedasticity-consistent standard errors were used for W-DEQ-A models, whereas conventional ordinary least-squares inference was used for PAI models.

Categorical predictors were dummy coded, with nulliparity, university education, employment, income equal to expenses, and planned pregnancy as reference categories. Pregnancy planning status was modeled as a three-category variable: planned pregnancy, unplanned pregnancy, and pregnancy following fertility treatment. Planned pregnancy was used as the reference category, with separate dummy variables representing unplanned pregnancy and pregnancy following fertility treatment. Multicollinearity was evaluated using tolerance and variance inflation factor (VIF) values; all VIF values were below 1.32. Heteroskedasticity was assessed using the Breusch–Pagan test. Because heteroskedasticity was detected in the W-DEQ-A model (*p* = 0.027), HC3 heteroskedasticity-consistent standard errors, 95% confidence intervals, and *p*-values were used for inference. The corresponding test was not statistically significant for the PAI model (*p* = 0.076), for which conventional ordinary least-squares inference was retained.

Several sensitivity analyses were conducted to evaluate the robustness of the primary findings. First, ACE exposure was entered categorically (0, 1–3, and ≥4 ACEs), with 0 ACEs as the reference category, to examine potential differences across exposure groups. Second, potential nonlinearity in the association between gestational age and fear of childbirth was assessed by adding a centered quadratic gestational-age term to the primary W-DEQ-A model. All original covariates were retained, and HC3 robust standard errors were used. Model-based predicted W-DEQ-A scores were estimated across the observed gestational-age range (12–40 weeks), with the remaining covariates held at their sample means. To further assess the potential influence of gestational age on prenatal attachment, an additional sensitivity analysis was conducted by incorporating a centered quadratic gestational-age term into the fully adjusted PAI regression model. Gestational age was also examined categorically using three groups (12–13, 14–27, and 28–40 completed weeks). The 14–27-week group was used as the reference category because only seven participants were assessed at 12–13 weeks. An additional sensitivity analysis excluded these seven early-pregnancy observations. All models retained the remaining covariates specified in the primary analyses.

Additional sensitivity analyses examined the influence of early-pregnancy observations by refitting the W-DEQ-A regression model after excluding women at 12–13 weeks of gestation, excluding those before 20 weeks, and restricting the analysis to women at 28–40 weeks. The original covariate structure and HC3 robust inference were maintained in each model. Potentially influential observations were assessed using Cook’s distance, with values exceeding 4/n flagged for further examination. Regression diagnostics included examination of residual distribution, externally studentized residuals, leverage values, and Cook’s distance. Residual normality was additionally evaluated using the Jarque–Bera test. Given the observed departures from residual normality and evidence of heteroskedasticity in the W-DEQ-A model, HC3 heteroskedasticity-consistent standard errors were used for coefficient inference. Influential observations were examined through sensitivity analyses to assess the stability of the primary findings. The same residual diagnostics were applied to the PAI regression model. Residual distribution was examined using the Jarque–Bera test, and potentially influential observations were assessed using externally studentized residuals, leverage values, and Cook’s distance. An additional sensitivity analysis was performed by refitting the PAI model after excluding observations exceeding the Cook’s distance screening threshold of 4/n. The model was refitted after excluding flagged observations to assess the stability of the ACE association. These analyses were interpreted as sensitivity checks rather than alternative primary models. All statistical tests were two-sided, and *p* < 0.05 was considered statistically significant.

### 2.7. Use of Generative Artificial Intelligence

During manuscript preparation, the authors used ChatGPT (OpenAI, GPT-5.6 Sol) for language editing, manuscript restructuring, assistance with statistical code generation, and document formatting. All AI-assisted outputs were reviewed and verified by the authors, who retain full responsibility for the accuracy, integrity, and interpretation of the manuscript.

## 3. Results

A total of 537 pregnant women were included, and all had valid data for the variables used in the primary analyses after source verification. Mean maternal age was 28.1 ± 4.47 years (range 18–43). Gestational age ranged from 12 to 40 completed weeks (mean ± SD: 31.12 ± 6.38). Seven participants (1.3%) were at 12–13 weeks, 128 (23.8%) at 14–27 weeks, and 402 (74.9%) at 28–40 weeks of gestation. Of the participants, 43.8% had university education, 35.2% high school education, 12.3% middle school education, 6.7% primary school education, and 2.0% postgraduate education. Overall, 36.9% were employed and 89.4% lived in nuclear families. Most pregnancies were planned (74.3%), 23.6% were unplanned, and 2.0% occurred following fertility treatment. Regarding parity, 259 women (48.2%) were nulliparous and 278 (51.8%) were multiparous.

The overall mean ACE score was 1.03 ± 1.42 (range: 0–10), the mean W-DEQ-A score was 66.10 ± 22.52 (range: 5–155), and the mean PAI score was 66.54 ± 9.88 (range: 25–84). Regarding ACE exposure, 248 participants (46.2%) reported no ACEs, 254 (47.3%) reported 1–3 ACEs, and 35 (6.5%) reported four or more ACEs.

### 3.1. Scale Scores by Sociodemographic Characteristics

ACE and W-DEQ-A scores did not differ significantly by educational level (Welch F(4,60.44) = 2.22, *p* = 0.077, ω^2^ = 0.001 and Welch F(4,58.81) = 0.50, *p* = 0.732, ω^2^ < 0.001, respectively). PAI scores differed significantly by education (Welch F(4,58.74) = 8.79, *p* < 0.001, ω^2^ = 0.061). Employed women had higher ACE and PAI scores than unemployed women, although the effect sizes were small (ACE: *p* = 0.009, |*r_rb_*| = 0.127; PAI: *p* < 0.001, |*r_rb_*| = 0.205). W-DEQ-A scores did not differ significantly by employment status (*p* = 0.059, |*r_rb_*| = 0.098). No statistically significant differences in the three scale scores were observed according to economic status or family type (Table 1).

### 3.2. Fear of Childbirth and Prenatal Attachment Across ACE Groups

W-DEQ-A scores differed significantly across ACE groups (Welch F = 13.90, *p* < 0.001, ω^2^ = 0.047). Women reporting no ACEs had lower W-DEQ-A scores than women reporting 1–3 ACEs (*p* < 0.001; Hedges’ g = 0.45) and ≥4 ACEs (*p* = 0.006; g = 0.49). The 1–3 and ≥4 ACE groups did not differ significantly (*p* = 0.889; g = 0.08). PAI scores did not differ significantly across ACE groups (χ^2^ = 5.13, *p* = 0.077, ε^2^ = 0.006) (Table 2).

### 3.3. Correlations Among Gestational Age, ACEs, Fear of Childbirth, and Prenatal Attachment

ACE total score was weakly positively correlated with W-DEQ-A score (ρ = 0.232, *p* < 0.001). The correlation between ACE and PAI scores was very weak (ρ = 0.092, *p* = 0.033) and did not persist in the primary multivariable model. W-DEQ-A and PAI scores were not significantly correlated (ρ = 0.013, *p* = 0.768). Gestational age was inversely correlated with W-DEQ-A score (ρ = −0.354, *p* < 0.001) and weakly inversely correlated with ACE score (ρ = −0.128, *p* = 0.003) (Table 3).

### 3.4. Multivariable Associations with Fear of Childbirth and Prenatal Attachment

In the minimally adjusted models controlling for maternal age and gestational age, ACE scores were positively associated with W-DEQ-A scores (B = 2.185, 95% CI: 0.919–3.450, *p* < 0.001; R^2^ = 0.127), whereas the association with PAI scores was not statistically significant (B = 0.276, 95% CI: −0.314–0.866, *p* = 0.358; R^2^ = 0.010). In the fully adjusted models, the corresponding ACE coefficients were 2.430 for W-DEQ-A (95% CI: 1.102–3.758, *p* < 0.001) and 0.375 for PAI (95% CI: −0.207–0.956, *p* = 0.206). The direction of the associations and their statistical significance were consistent across the two adjustment specifications. A comparison of minimally and fully adjusted regression models is provided in Supplementary Table S1.

**Figure 1:**
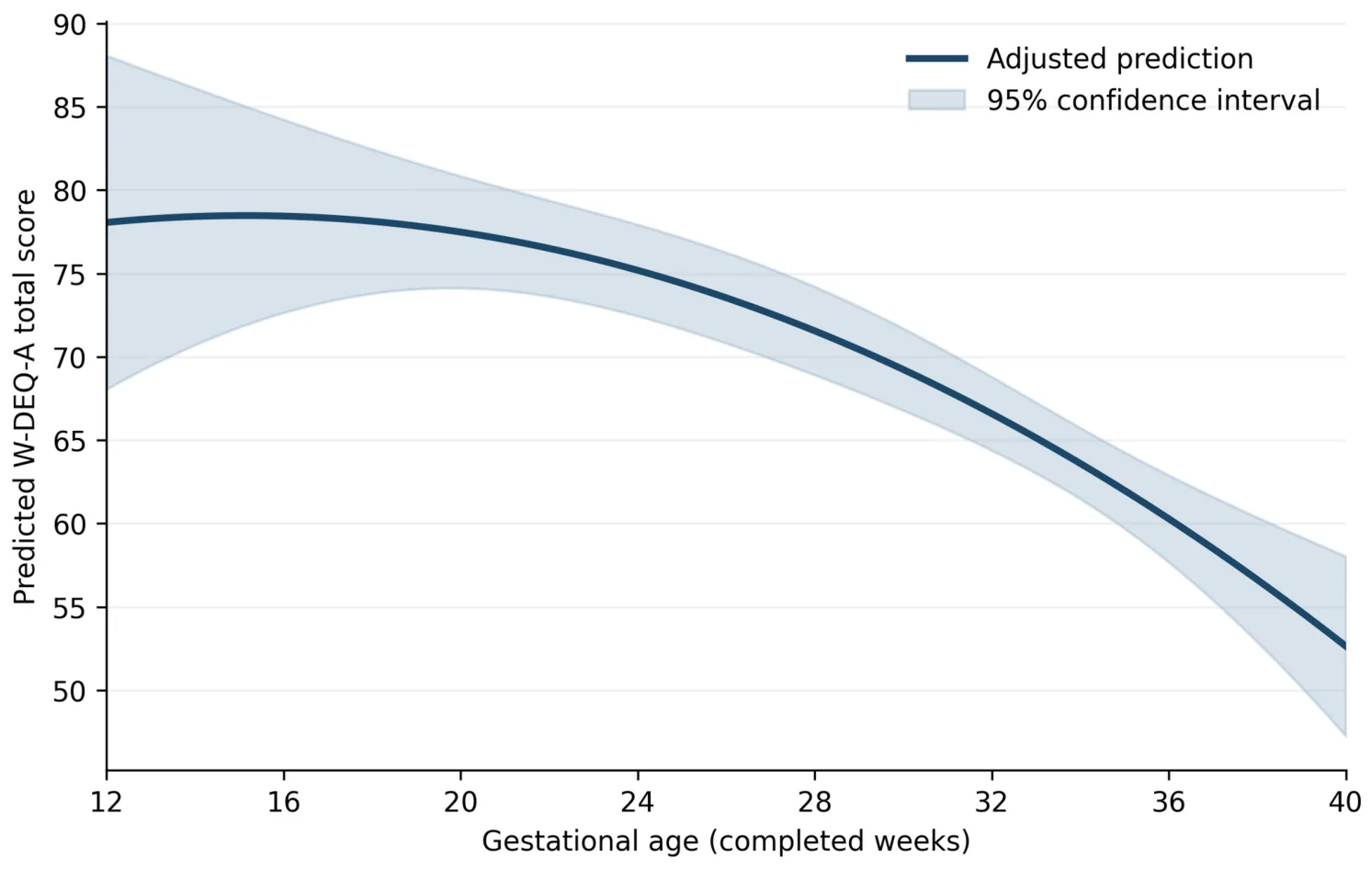
Adjusted predicted fear-of-childbirth scores across gestational age from the quadratic regression model. Note: The solid line represents model-based predicted W-DEQ-A scores, and the shaded area indicates 95% confidence intervals. Predictions were calculated with other covariates held at their sample means. The model included adverse childhood experiences, maternal age, gestational age, parity, educational level, employment status, economic status, and pregnancy planning status. HC3 heteroskedasticity-consistent standard errors were used. The estimated association reflects between-participant differences and should not be interpreted as within-person changes across pregnancy.

The W-DEQ-A regression model included all 537 women and explained 14.7% of the variance in fear-of-childbirth scores (R^2^ = 0.147, adjusted R^2^ = 0.126). Because heteroskedasticity was detected, coefficient inference was based on HC3 robust standard errors. Higher ACE total scores were associated with higher W-DEQ-A scores after adjustment for measured covariates (B = 2.430, β = 0.154, 95% CI: 1.102–3.758, *p* < 0.001). Gestational age was inversely associated with W-DEQ-A scores in the primary linear model (B = −1.057, β = −0.299, 95% CI: −1.345–−0.770, *p* < 0.001). Compared with women reporting income equal to expenses, those reporting income greater than expenses had lower W-DEQ-A scores (B = −7.118, β = −0.092, 95% CI: −13.937–−0.300, *p* = 0.041). This secondary finding should be interpreted cautiously given the number of covariates examined and the absence of multiplicity adjustment. The remaining covariates were not statistically significant. In the categorical ACE sensitivity model, compared with women reporting no ACEs, both women reporting 1–3 ACEs (B = 7.702, 95% CI: 3.784–11.619, *p* < 0.001) and those reporting ≥4 ACEs (B = 11.608, 95% CI: 4.413–18.803, *p* = 0.002) had higher W-DEQ-A scores. However, the adjusted difference between the 1–3 and ≥4 ACE groups was not statistically significant (*p* = 0.274). In the quadratic gestational-age sensitivity model, the centered squared gestational-age term was statistically significant (B = −0.042, 95% CI: −0.083–−0.0004, *p* = 0.048), with an R^2^ of 0.154. The model suggested a relatively flat association during early pregnancy, followed by progressively lower predicted fear-of-childbirth scores at later gestational weeks. The adjusted predicted W-DEQ-A scores were 77.48 at 20 weeks, 66.58 at 32 weeks, and 52.63 at 40 weeks. The positive association between ACE scores and fear of childbirth remained statistically significant (B = 2.477, 95% CI: 1.155–3.798, *p* < 0.001). The quadratic model retained all covariates from the primary regression model and used HC3 robust standard errors (Figure 1).

Additional sensitivity analyses showed that the positive association between ACE scores and fear of childbirth remained statistically significant after excluding women at 12–13 weeks of gestation (n = 530; B = 2.513, 95% CI: 1.168–3.859, *p* < 0.001), excluding those before 20 weeks (n = 498; B = 2.424, 95% CI: 1.022–3.825, *p* < 0.001), and restricting the analysis to women at 28–40 weeks (n = 402; B = 2.683, 95% CI: 1.117–4.249, *p* < 0.001). Regression diagnostics indicated deviations from residual normality (Jarque–Bera test, *p* < 0.001). Three observations had absolute externally studentized residuals exceeding 3, and the maximum leverage value was 0.119. Cook’s distance exceeded the 4/n screening threshold for 32 observations, although none exceeded 1. After excluding these flagged observations, the ACE association remained statistically significant (n = 505; B = 2.652, 95% CI: 1.724–3.581, *p* < 0.001). These sensitivity analyses suggest that the association was not driven solely by early-pregnancy observations or potentially influential cases. Additional sensitivity analyses for fear of childbirth are summarized in Supplementary Table S2.

The fully adjusted PAI regression model included all 537 women and explained 11.9% of the variance in prenatal attachment scores (R^2^ = 0.119, adjusted R^2^ = 0.097; Table 4). ACE total score was not significantly associated with PAI after adjustment for measured covariates (B = 0.375, β = 0.054, 95% CI: −0.207–0.956, *p* = 0.206). In secondary exploratory analyses, educational level was associated with PAI scores overall (F(4, 523) = 5.27, *p* < 0.001). Compared with university graduates, women with middle school (B = −5.079, *p* < 0.001) and high school education (B = −2.798, *p* = 0.004) had lower PAI scores. Unplanned pregnancy was also associated with lower PAI scores than planned pregnancy (B = −3.905, β = −0.168, 95% CI: −5.855–−1.955, *p* < 0.001). Employment status was not significantly associated with PAI after adjustment (*p* = 0.144). These secondary findings should be interpreted cautiously because multiple regression coefficients were examined without formal adjustment for multiplicity.

In the exploratory categorical ACE sensitivity analysis, the overall association between ACE categories and prenatal attachment was not statistically significant (F(2, 522) = 2.47, *p* = 0.086). Although women reporting 1–3 ACEs showed a nominally significant positive contrast compared with women reporting no ACEs (B = 1.806, 95% CI: 0.124–3.487, unadjusted *p* = 0.035), the ≥4 ACE group did not differ significantly from the reference group (B = −0.240, 95% CI: −3.654–3.174, *p* = 0.890). Given the nonsignificant overall test, multiple exploratory comparisons, and non-monotonic pattern, the isolated nominally significant contrast should not be interpreted as evidence of a consistent or clinically meaningful positive association between ACE exposure and prenatal attachment.

Regression diagnostics for the PAI model indicated deviations from residual normality (Jarque–Bera test, *p* < 0.001). Three observations had absolute externally studentized residuals exceeding 3, and the maximum leverage value was 0.119. Cook’s distance exceeded the 4/n screening threshold for 33 observations, although none exceeded 1 (maximum = 0.075). After excluding the flagged observations, the ACE–PAI association became statistically significant in the exploratory sensitivity analysis (B = 0.769, *p* = 0.006), unlike in the primary adjusted model. Because Cook’s distance identifies potentially influential rather than necessarily erroneous observations, this result indicates sensitivity to influential cases and should not be interpreted as evidence of a robust positive association.

Additional sensitivity analyses examined the possibility of a nonlinear association between gestational age and prenatal attachment. The centered quadratic gestational-age term was statistically significant (B = −0.023, 95% CI: −0.041–−0.006, *p* = 0.009; R^2^ = 0.131). The association between ACE scores and prenatal attachment remained nonsignificant in the quadratic model (B = 0.401, 95% CI: −0.177–0.979, *p* = 0.174). When gestational age was modeled categorically (12–13, 14–27, and 28–40 completed weeks), the overall group effect was not statistically significant (F(2, 522) = 1.11, *p* = 0.331). Excluding the seven women at 12–13 weeks of gestation did not materially change the ACE–PAI association (n = 530; B = 0.341, 95% CI: −0.245–0.927, *p* = 0.253). Although the quadratic model suggested potential nonlinearity, the unequal distribution of gestational ages and the small early-pregnancy subgroup warrant cautious interpretation. Overall, the ACE–PAI association remained statistically nonsignificant across these gestational-age sensitivity analyses. Detailed results of the gestational-age sensitivity analyses for prenatal attachment are presented in Supplementary Table S3.

**Table 4:** Multivariable linear regression models for fear of childbirth and prenatal attachment.

Outcome/Predictor	B	SE	β	95% CI	*p*
**W-DEQ-A model**					
ACE total	2.430	0.678	0.154	1.102–3.758	<0.001
Maternal age	−0.319	0.253	−0.063	−0.816–0.177	0.207
Gestational age	−1.057	0.147	−0.299	−1.345–−0.770	<0.001
Multiparity	2.784	2.081	0.062	−1.295–6.862	0.181
Education: Primary	1.361	4.787	0.015	−8.021–10.743	0.776
Education: Middle school	−2.912	2.972	−0.042	−8.736–2.913	0.327
Education: High school	1.445	2.214	0.031	−2.895–5.785	0.514
Education: Postgraduate	5.978	6.393	0.038	−6.552–18.509	0.350
Unemployed	−0.554	2.133	−0.012	−4.735–3.627	0.795
Income < expenses	−1.811	2.498	−0.033	−6.708–3.085	0.468
Income > expenses	−7.118	3.479	−0.092	−13.937–−0.300	0.041
Unplanned pregnancy	−3.956	2.428	−0.075	−8.715–0.802	0.103
Fertility treatment	2.969	5.944	0.019	−8.680–14.619	0.617
**PAI model**					
ACE total	0.375	0.296	0.054	−0.207–0.956	0.206
Maternal age	0.191	0.102	0.086	−0.009–0.390	0.061
Gestational age	0.054	0.065	0.035	−0.074–0.182	0.406
Multiparity	−1.432	0.917	−0.073	−3.233–0.369	0.119
Education: Primary	−0.205	1.751	−0.005	−3.644–3.234	0.907
Education: Middle school	−5.079	1.364	−0.169	−7.759–−2.399	<0.001
Education: High school	−2.798	0.972	−0.135	−4.707–−0.889	0.004
Education: Postgraduate	4.253	2.978	0.061	−1.596–10.103	0.154
Unemployed	−1.359	0.928	−0.066	−3.182–0.464	0.144
Income < expenses	−0.735	1.051	−0.031	−2.800–1.329	0.484
Income > expenses	−0.508	1.450	−0.015	−3.356–2.340	0.726
Unplanned pregnancy	−3.905	0.993	−0.168	−5.855–−1.955	<0.001
Fertility treatment	3.537	2.954	0.051	−2.266–9.340	0.232

Note: B, unstandardized regression coefficient; SE, standard error; β, standardized regression coefficient; CI, confidence interval; ACE, adverse childhood experiences; W-DEQ-A, Wijma Delivery Expectancy/Experience Questionnaire-A; PAI, Prenatal Attachment Inventory. Reference categories: nulliparity, university education, employed, income equal to expenses, and planned pregnancy. W-DEQ-A: HC3 heteroskedasticity-consistent SE, 95% CI and *p* values; n = 537, R^2^ = 0.147, adjusted R^2^ = 0.126. PAI: conventional OLS inference; n = 537, R^2^ = 0.119, adjusted R^2^ = 0.097.

## 4. Discussion

This study examined ACEs in relation to fear of childbirth and prenatal attachment within the same sample while accounting for relevant sociodemographic and obstetric characteristics. The principal finding was that higher ACE scores were associated with greater fear of childbirth after adjustment for measured sociodemographic and obstetric characteristics, and this association remained statistically significant across sensitivity analyses. In contrast, the very weak bivariate association between ACEs and prenatal attachment did not persist after multivariable adjustment. In secondary exploratory analyses, educational level and pregnancy planning status were associated with prenatal attachment after adjustment for the measured covariates; however, these findings should be interpreted cautiously because no multiplicity adjustment was applied.

Each one-point increase in ACE total score was associated with an approximately 2.43-point increase in W-DEQ-A score after adjustment for the measured covariates in the primary model. This finding is consistent with previous research reporting associations between childhood trauma and fear of childbirth [7,8,18] and with broader evidence linking childhood adversity to psychological vulnerability during the perinatal period [19]. Earlier traumatic experiences may heighten sensitivity to themes of bodily exposure, control, safety, and anticipated medical intervention during childbirth [6,20]. Nevertheless, because the present data are cross-sectional, the findings should be interpreted as associations rather than evidence that ACEs cause fear of childbirth. Although the association between ACE scores and fear of childbirth persisted after adjustment for the measured sociodemographic and obstetric characteristics, this finding should not be interpreted as evidence of an independent causal effect. Maternal psychological factors, including depressive symptoms, anxiety, post-traumatic stress symptoms, previous mental-health history, and perceived social support, may be associated with both childhood adversity and childbirth-related fear. Previous birth experiences may also contribute to these relationships. As these factors were not comprehensively assessed in the present study, residual confounding cannot be excluded. Furthermore, some psychological factors may function as mediators rather than confounders, and their roles cannot be distinguished using the current cross-sectional data.

The association between ACE scores and fear of childbirth remained statistically significant in both minimally and fully adjusted models. However, the distinction between these models is conceptually important. Educational attainment, employment, economic circumstances, reproductive history, and pregnancy planning may occur after childhood adversity and could potentially lie along pathways linking ACEs to maternal psychological outcomes. Adjustment for such variables may therefore alter the estimated association through overadjustment or other sources of bias. The consistency of the ACE association across model specifications is reassuring, but neither model can establish a causal effect. Given the observational and cross-sectional design, the causal roles of these variables remain uncertain.

The categorical sensitivity analysis provided further insight into the association between ACE exposure and fear of childbirth. Compared with women reporting no ACEs, those reporting 1–3 ACEs and ≥4 ACEs had adjusted W-DEQ-A scores approximately 7.7 and 11.6 points higher, respectively. However, the difference between the two ACE-exposed groups was not statistically significant, providing insufficient evidence for a graded dose–response relationship across these categories. Furthermore, the ≥4 ACE group was relatively small (n = 35), limiting the precision of estimates for women with high cumulative adversity exposure. Considering the W-DEQ-A score range of 0–165, the observed adjusted differences should be interpreted cautiously. Although statistically significant, their clinical importance cannot be established without an appropriately validated threshold or clinically meaningful difference for this population. These findings therefore support an association between reported childhood adversity and greater fear-of-childbirth scores rather than a definitive dose–response pattern or clinically predictive relationship.

The association between gestational age and fear of childbirth appeared to be nonlinear, with the quadratic model suggesting relatively stable predicted scores during early pregnancy and lower scores at later gestational weeks. However, this pattern should be interpreted cautiously, particularly because relatively few participants were assessed during early pregnancy. Importantly, the cross-sectional design precludes any inference that childbirth-related fear decreases within individual women as pregnancy progresses. Longitudinal studies with repeated measurements are needed to clarify the temporal pattern of fear of childbirth across gestation. The association between ACE scores and fear of childbirth remained statistically significant after accounting for the quadratic gestational-age term.

For prenatal attachment, the quadratic gestational-age model suggested a potentially nonlinear association, although the categorical gestational-age comparison did not identify significant overall differences. These findings should be interpreted cautiously because the sample was heavily concentrated in late pregnancy, with only seven participants assessed at 12–13 weeks. Moreover, the cross-sectional design and unequal group sizes limit conclusions about changes in prenatal attachment across pregnancy. Importantly, the association between ACE scores and prenatal attachment remained statistically nonsignificant across the additional gestational-age sensitivity analyses.

The primary multivariable model did not identify a statistically significant association between ACE scores and prenatal attachment after adjustment for measured sociodemographic and obstetric characteristics. Although the exploratory categorical sensitivity analysis yielded a nominally significant positive contrast for women reporting 1–3 ACEs compared with those reporting no ACEs, the overall ACE-category effect was not statistically significant. Furthermore, the absence of a corresponding association in the ≥4 ACE group and the non-monotonic pattern do not support a consistent relationship between cumulative childhood adversity and prenatal attachment. Given the multiple exploratory comparisons and the lack of adjustment for multiplicity, this isolated finding may reflect chance and should not be considered evidence of a clinically meaningful positive association. Previous research suggests that associations between childhood adversity and maternal–fetal attachment may involve maternal mental health, attachment patterns, and perceived social support [9,10,11,21]. However, these potential pathways were not comprehensively assessed in the present study, and their roles could not be examined. Future longitudinal research incorporating these psychosocial factors may help clarify the relationship between childhood adversity and prenatal attachment.

Educational level and pregnancy planning status were significantly associated with prenatal attachment after adjustment for the measured covariates. Compared with university graduates, women with middle school and high school education had lower PAI scores. Educational level may reflect differences in health literacy, access to pregnancy-related information, or other social resources, but these mechanisms were not directly measured. In addition, unplanned pregnancy was associated with an approximately four-point lower PAI score than planned pregnancy. The cross-sectional design precludes conclusions about direction or mechanism, and these findings should therefore be interpreted as adjusted associations rather than causal effects.

Employment status illustrates the importance of adjusted analysis. Although employed women had higher prenatal attachment scores in unadjusted comparisons, employment status was no longer statistically significantly associated with PAI scores after adjustment for the measured covariates. The unadjusted difference may therefore reflect shared variance with education, economic circumstances, pregnancy planning, or other characteristics rather than an independent association.

W-DEQ-A and PAI scores were not significantly correlated in this sample. This suggests that fear of childbirth and prenatal attachment may capture partly distinct aspects of prenatal psychological experience. However, the absence of a statistical correlation should not be interpreted as evidence that the two psychological processes are completely independent.

### Strengths and Limitations

This study has several strengths, including its relatively large sample size, the simultaneous assessment of fear of childbirth and prenatal attachment, and the use of theoretically informed multivariable regression models. The reporting of effect sizes, regression diagnostics, and sensitivity analyses further strengthens the analytical approach. HC3 heteroskedasticity-consistent inference, alternative ACE exposure classifications, examination of potential nonlinearity in gestational age, and influential-observation analyses allowed a more comprehensive evaluation of the stability of the findings. Nevertheless, several limitations should be considered. First, the cross-sectional design precludes causal and temporal inferences. Although gestational age was associated with fear of childbirth, the observed pattern cannot be interpreted as evidence of changes in fear within individual women as pregnancy progresses. The quadratic model suggested potential nonlinearity, but the relatively small number of participants assessed during early pregnancy limits the precision of this finding. Although the W-DEQ-A and PAI were administered during pregnancy, the present study did not evaluate whether their measurement properties were invariant across gestational-age groups. Consequently, potential differences in how these instruments function across pregnancy cannot be excluded. The relatively small number of participants assessed at 12–13 weeks further limits interpretation of early-pregnancy findings and the precision of comparisons across gestational periods. Longitudinal studies involving repeated assessments are needed to clarify these associations. Second, adverse childhood experiences were assessed retrospectively and may have been affected by recall bias. Although data were collected in a private setting and participants were informed that they could decline to answer individual questions, face-to-face administration of sensitive ACE items may have contributed to social-desirability bias, reluctance to disclose adverse experiences, or underreporting. Furthermore, the ACE scale demonstrated limited internal consistency (Cronbach’s α = 0.641). Although ACE scores represent a cumulative index of heterogeneous adverse experiences rather than a unidimensional psychological construct, potential measurement error cannot be excluded. Third, the use of nonprobability convenience sampling at a single healthcare institution limits the generalizability of the findings. Eligibility was restricted to women living with a spouse and those without clinically significant previous or current obstetric complications. Although these criteria were established to reduce the potential influence of difficult or traumatic obstetric experiences, they may have resulted in the underrepresentation of women with higher levels of childbirth-related fear. Consequently, the observed distribution of fear-of-childbirth scores and their association with ACE exposure may differ in more heterogeneous obstetric populations. The number of women who did not meet the eligibility criteria was not systematically recorded, preventing quantification of potential selection bias. Additionally, information on obstetric complications was based on self-report rather than independent verification of medical records. Fourth, the relatively small number of women reporting four or more ACEs (n = 35) limited the precision of high-exposure comparisons. Although higher ACE scores were associated with greater fear of childbirth, the categorical analyses did not provide convincing evidence of a graded dose–response relationship. Furthermore, the clinical significance of the observed W-DEQ-A score differences remains uncertain, and statistical significance should not be interpreted as evidence of clinical importance. Fifth, potentially relevant psychosocial and clinical factors, including depressive symptoms, anxiety, post-traumatic stress symptoms, previous mental-health history, perceived social support, and detailed previous childbirth experiences, were not comprehensively assessed or included in the regression models. Their omission may have resulted in residual confounding. Some of these factors may also operate as mediators, but their potential roles could not be evaluated using the available cross-sectional data. Therefore, the findings should be interpreted as associations adjusted for measured covariates rather than evidence of independent causal effects. Importantly, the exclusion of women with current or previous clinically significant obstetric complications may have introduced selection bias beyond limiting generalizability. If childhood adversity is associated with the likelihood of obstetric complications, and these complications are also associated with fear of childbirth, restricting the sample to women without such complications could alter the observed ACE–fear-of-childbirth association. The direction and magnitude of this potential selection bias cannot be determined from the available data. Consequently, the observed association should be interpreted within the context of the study’s eligibility criteria and should not be assumed to apply to women experiencing obstetric complications.

Finally, regression diagnostics identified departures from residual normality and potentially influential observations. Although the association between ACE scores and fear of childbirth remained statistically significant across sensitivity analyses excluding early-pregnancy observations and observations flagged by Cook’s distance, the prenatal attachment findings were more sensitive to influential observations. Specifically, the ACE–PAI association was not statistically significant in the primary adjusted model but became significant after excluding observations exceeding the Cook’s distance screening threshold. Because this exclusion was exploratory and the flagged observations were not necessarily erroneous, the resulting association should not be interpreted as robust evidence of a positive relationship between ACE exposure and prenatal attachment. This sensitivity highlights uncertainty in the prenatal attachment findings and reinforces the importance of prioritizing the prespecified primary model. Additionally, the multiple exploratory comparisons conducted without formal multiplicity adjustment increase the possibility of chance findings. Taken together, these limitations emphasize the need for cautious interpretation and highlight the importance of longitudinal, multicenter studies incorporating more comprehensive psychosocial assessments and diverse obstetric populations.

## 5. Conclusions

Higher ACE scores were associated with greater fear of childbirth after adjustment for measured sociodemographic and obstetric characteristics. This association remained statistically significant across sensitivity analyses; however, the categorical findings did not provide convincing evidence of a graded dose–response relationship, and the clinical significance of the observed differences remains uncertain. ACE scores were not significantly associated with prenatal attachment in the primary adjusted model, although sensitivity analyses indicated some instability in this finding. Secondary exploratory analyses also identified associations of educational level and pregnancy planning status with prenatal attachment, although these findings require confirmation in future studies. These results highlight the potential relevance of trauma-informed, privacy-sensitive antenatal care but do not establish causality or justify universal trauma screening. Further longitudinal and multicenter studies incorporating maternal mental health, social support, and diverse obstetric populations are warranted.

## Figures and Tables

**Table 1:** Scale scores according to sociodemographic characteristics.

Variable	*n* (%)	ACE Mean ± SD	ACE *p*/ES	W-DEQ-A Mean ± SD	W-DEQ-A *p*/ES	PAI Mean ± SD	PAI *p*/ES
**Education**							
Primary school	36 (6.7)	0.58 ± 0.91	0.077/ω^2^ = 0.001	65.86 ± 29.34	0.732/ω^2^ < 0.001	67.08 ± 8.75	<0.001/ω^2^ = 0.061
Middle school	66 (12.3)	1.15 ± 1.63		62.74 ± 20.14		62.30 ± 11.35	
High school	189 (35.2)	0.99 ± 1.39		66.77 ± 23.25		64.71 ± 9.73	
University	235 (43.8)	1.10 ± 1.45		66.49 ± 21.59		68.82 ± 9.02	
Postgraduate	11 (2.0)	1.00 ± 1.26		67.09 ± 19.62		73.18 ± 9.02	
**Employment**							
Employed	198 (36.9)	1.20 ± 1.49	0.009/*r_rb_* = 0.127	68.12 ± 21.31	0.059/*r_rb_* = 0.098	68.65 ± 9.86	<0.001/*r_rb_* = 0.205
Unemployed	339 (63.1)	0.93 ± 1.38		64.91 ± 23.15		65.32 ± 9.69	
**Economic status**							
Income < expenses	115 (21.4)	1.22 ± 1.70	0.309/ω^2^ = 0.002	64.96 ± 23.23	0.058/ω^2^ = 0.007	64.60 ± 10.00	0.053/ω^2^ = 0.008
Income = expenses	372 (69.3)	0.96 ± 1.32		67.33 ± 22.28		66.96 ± 9.79	
Income > expenses	50 (9.3)	1.10 ± 1.49		59.58 ± 21.82		67.96 ± 9.80	
**Family type**							
Nuclear	480 (89.4)	1.01 ± 1.37	0.719/*r_rb_* = 0.027	66.39 ± 21.97	0.428/*r_rb_* = 0.064	66.59 ± 9.81	0.651/*r_rb_* = 0.037
Extended	57 (10.6)	1.19 ± 1.79		63.60 ± 26.82		66.16 ± 10.46	
**Parity**							
Nulliparous	259 (48.2)	1.08 ± 1.49	0.949/r = 0.003	65.29 ± 23.24	0.545/r = 0.030	67.39 ± 9.79	0.030/r = 0.108
Multiparous	278 (51.8)	0.99 ± 1.36		66.85 ± 21.85		65.74 ± 9.91	

Note: Means and standard deviations are presented for descriptive comparability. Inferential tests were selected according to distributional and variance assumptions. ES, effect size; *r_rb_*, rank-biserial correlation.

**Table 2:** W-DEQ-A and PAI scores according to ACE category.

Outcome	0 ACEs (*n* = 248)	1–3 ACEs (*n* = 254)	≥4 ACEs (*n* = 35)	Test	*p*	Effect Size
W-DEQ-A	60.63 ± 24.14	70.59 ± 20.04	72.20 ± 19.15	Welch F = 13.90	<0.001	ω^2^ = 0.047
PAI	65.73 ± 9.38	67.44 ± 9.97	65.69 ± 12.17	χ^2^ = 5.13	0.077	ε^2^ = 0.006

Note: ACE-group totals were 248, 254, and 35 for 0, 1–3, and ≥4 ACEs, respectively; all outcomes were available for all 537 participants. Games–Howell post hoc comparisons for W-DEQ-A: 0 vs. 1–3 ACEs, *p* < 0.001, g = 0.45; 0 vs. ≥4 ACEs, *p* = 0.006, g = 0.49; 1–3 vs. ≥4 ACEs, *p* = 0.889, g = 0.08.

**Table 3:** Spearman correlations among gestational age, ACE, W-DEQ-A, and PAI scores.

Variable	1	2	3	4
1. Gestational age	1			
2. ACE total	−0.128 **	1		
3. W-DEQ-A total	−0.354 ***	0.232 ***	1	
4. PAI total	−0.063	0.092 *	0.013	1

Note: * *p* < 0.05; ** *p* < 0.01; *** *p* < 0.001. All correlations were based on n = 537.

## Data Availability

The data supporting the findings of this study are available from the corresponding author upon reasonable request. The dataset is not publicly available because it contains sensitive psychosocial information, including adverse childhood experiences, and public sharing was not included in the original participant consent.

## Supplementary Materials

The following supporting information can be downloaded at: https://www.mdpi.com/article/10.3390/healthcare14213662/s1, Table S1: Comparison of minimally and fully adjusted associations of ACE total score with W-DEQ-A and PAI; Table S2: Additional sensitivity analyses for the ACE association with fear of childbirth (W-DEQ-A); Table S3: Gestational-age sensitivity analyses for prenatal attachment (PAI).

## Abbreviations

The following abbreviations are used in this manuscript:

ACE	Adverse childhood experiences
PAI	Prenatal Attachment Inventory
W-DEQ-A	Wijma Delivery Expectancy/Experience Questionnaire-A
VIF	Variance inflation factor
