# Supplementary material for: Association of Adverse Childhood Experiences with Fear of Childbirth and Prenatal Attachment Among Pregnant Women: A Cross-Sectional Study
Source: Healthcare (Basel). 2026 Oct 28;14(21):3662. doi: 10.3390/healthcare14213662 (PMC13762743; doi:10.3390/healthcare14213662)
Supplement: Supplementary file 1 [file healthcare-14-03662-s001.zip › healthcare-4625243-supplementary.pdf]

Supplementary Materials

Association of Adverse Childhood Experiences with Fear of Childbirth and Prenatal Attachment Among Pregnant Women: A Cross-Sectional Study

All estimates below were reproduced using the corrected n = 537 dataset (537(8).xlsx), using the model coding described in the manuscript. Values are rounded for presentation.

Table S1. Comparison of minimally and fully adjusted associations of ACE total score with W-DEQ-A and PAI.

Outcome/model	B	SE	95% CI	p	R^2
**W-DEQ-A, minimally adjusted**	2.185	0.646	0.919–3.450	<0.001	0.127
**W-DEQ-A, fully adjusted**	2.430	0.678	1.102–3.758	<0.001	0.147
**PAI, minimally adjusted**	0.276	0.300	−0.314–0.866	0.358	0.010
**PAI, fully adjusted**	0.375	0.296	−0.207–0.956	0.206	0.119

Note: Coefficients represent the association with a one-point higher ACE total score. Minimally adjusted models include ACE total score, maternal age and gestational age. Fully adjusted models additionally include parity, educational level, employment status, economic status and pregnancy planning status. W-DEQ-A: HC3 robust standard errors; PAI: conventional OLS inference. All n = 537. The models quantify conditional associations, not causal effects.

Table S2. Additional sensitivity analyses for the ACE association with fear of childbirth (W-DEQ-A).

Analysis	n	ACE B	95% CI	p
**Fully adjusted linear model**	537	2.430	1.102–3.758	<0.001
**Quadratic gestational-age model**	537	2.477	1.155–3.798	<0.001
**Excluded 12–13 weeks**	530	2.513	1.168–3.859	<0.001
**Excluded <20 weeks**	498	2.424	1.022–3.825	<0.001
**Restricted to 28–40 weeks**	402	2.683	1.117–4.249	<0.001
**Excluded Cook's distance >4/n**	505	2.652	1.724–3.581	<0.001

Note: All models adjusted for maternal age, gestational age (with the added quadratic term only in the quadratic model), parity, educational level, employment status, economic status and pregnancy planning status, and used HC3 robust inference. Cook's distance identifies potentially influential rather than necessarily erroneous observations.

Table S3. Gestational-age sensitivity analyses for prenatal attachment (PAI).

Analysis	n	Parameter	B (95% CI)	p	R^2
**Primary linear model**	537	ACE total	0.375 (−0.207–0.956)	0.206	0.119
**Quadratic model**	537	ACE total	0.401 (−0.177–0.979)	0.174	0.131
**Quadratic model**	537	Gestational age²	−0.023 (−0.041–−0.006)	0.009	0.131
**Excluded 12–13 weeks**	530	ACE total	0.341 (−0.245–0.927)	0.253	0.110

Note: Quadratic gestational-age term is centered on mean gestational age. All models include the fully adjusted covariates and use conventional OLS inference. In the categorical gestational-age sensitivity analysis (12–13, 14–27 and 28–40 completed weeks), the joint group effect was $F(2, 522) = 1.11$, $p = 0.331$. Group sizes were 7, 128 and 402, respectively. The early-pregnancy subgroup was small and group estimates require caution. Abbreviations: ACE, adverse childhood experiences; W-DEQ-A, Wijma Delivery Expectancy/Experience Questionnaire-A; PAI, Prenatal Attachment Inventory; B, unstandardized regression coefficient; SE, standard error; CI, confidence interval; OLS, ordinary least squares.
